# Immunosignals of Oligodendrocyte Markers and Myelin-Associated Proteins Are Critically Affected after Experimental Stroke in Wild-Type and Alzheimer Modeling Mice of Different Ages

**DOI:** 10.3389/fncel.2018.00023

**Published:** 2018-02-06

**Authors:** Dominik Michalski, Anna L. Keck, Jens Grosche, Henrik Martens, Wolfgang Härtig

**Affiliations:** ^1^Department of Neurology, University of Leipzig, Leipzig, Germany; ^2^Paul Flechsig Institute for Brain Research, University of Leipzig, Leipzig, Germany; ^3^Effigos GmbH, Leipzig, Germany; ^4^Synaptic Systems GmbH, Göttingen, Germany

**Keywords:** oligodendrocyte, oligodendrocyte progenitor cells, myelin basic protein, stroke, cerebral ischemia, animal model, 3xTg mouse

## Abstract

Because stroke therapies are still limited and patients are often concerned by long-term sequelae with significant impairment of daily living, elaborated neuroprotective strategies are needed. During the last decades, research substantially improved the knowledge on cellular pathologies responsible for stroke-related tissue damage. In this context, the neurovascular unit (NVU) concept has been established, summarizing the affections of neurons, associated astrocytes and the vasculature. Although oligodendrocytes were already identified to play a major role in other brain pathologies, their role during stroke evolution and long-lasting tissue damage is poorly understood. This study aims to explore oligodendrocyte structures, i.e., oligodendrocytes and their myelin-associated proteins, after experimental focal cerebral ischemia. For translational issues, different ages and genotypes including an Alzheimer-like background were considered to mimic potential co-morbidities. Three- and 12-month-old wild-type and triple-transgenic mice were subjected to unilateral middle cerebral artery occlusion. Immunofluorescence labeling was performed on forebrain tissues affected by 24 h of ischemia to visualize the oligodendrocyte-specific protein (OSP), the myelin basic protein (MBP), and the neuron-glia antigen 2 (NG2) with reference to the ischemic lesion. Subsequent analyses concomitantly detected the vasculature and the 2′, 3′-cyclic nucleotide-3′-phosphodiesterase (CNPase) to consider the NVU concept and to explore the functional relevance of histochemical data on applied oligodendrocyte markers. While the immunosignal of NG2 was found to be nearly absent 24 h after ischemia onset, enhanced immunoreactivities for OSP and especially MBP were observed in close regional association to the vasculature. Added quantitative analyses based on inter-hemispheric differences of MBP-immunoreactivity revealed a shell-like pattern with a significant increase directly in the ischemic core, followed by a gradual decline toward the striatum, the ischemic border zone and the lateral neocortex. This observation was consistent in subsequent analyses on the potential impact of age and genetic background. Furthermore, immunoreactivities for CNPase, MBP, and OSP were found to be simultaneously enhanced. In conclusion, this study provides evidence for a critical role of oligodendrocyte structures in the early phase after experimental stroke, strengthening their involvement in the ischemia-affected NVU. Consequently, oligodendrocytes and their myelin-associated proteins may qualify as potential targets for neuroprotective and regenerative approaches in stroke.

## Introduction

Ischemic stroke still ranges among the three most common causes of death worldwide, and survivors are often concerned by long-term sequels like aphasia and functional relevant limb paresis, resulting in an enormous personal and socio-economic burden (Kolominsky-Rabas et al., 1998; Benjamin et al., 2017). Strategies for acute stroke treatment are currently focused on the recanalization of occluded cerebral vessels (Hankey, 2017), which can be facilitated by intravenous thrombolysis (Hacke et al., 2008) and – in the subpopulation of patients with large vessel occlusions – added mechanical thrombectomy (Goyal et al., 2016). However, as these strategies only reach the minority of acute stroke patients (Dirks et al., 2011), research was intensified toward the pathophysiology of stroke evolution and related tissue damage (Dirnagl et al., 1999) to explore potential neuroprotective treatments.

As a landmark of the emerging knowledge on stroke-related tissue damage, the concept of the neurovascular unit (NVU) describes the simultaneous affection and interplay of endothelial cells, associated astrocytes and pericytes under ischemic condition (del Zoppo, 2008, 2010; Xing et al., 2012). As endothelial structures are essentially involved in forming the blood – brain barrier (BBB), the NVU is critically linked to the maintenance of the BBB as well as its impaired integrity during ischemic stroke (Sandoval and Witt, 2008; Dyrna et al., 2013; Keaney and Campbell, 2015; Krueger et al., 2015). Moreover, the complexity of interactions within the NVU and the associated BBB might also help to understand the still existing translational roadblock, which means the lacking efficacy of preclinical well-established approaches under clinical conditions – as for instance shown for the free radical scavenger NXY-059 (Savitz, 2007; Endres et al., 2008).

A further population of neural cells are oligodendrocytes that became attraction due to their special role in forming myelin sheaths enwrapping neuronal axons (Simons and Nave, 2015; Komori, 2017; Philips and Rothstein, 2017). From a disease-related perspective, oligodendrocytes were identified to play a key role during development and maintenance of multiple sclerosis, where oligodendrocyte precursor cells fail to differentiate into mature oligodendrocytes with the property of myelin formation (Kotter et al., 2011; Mahad et al., 2015). Consequently, treatment strategies for multiple sclerosis focused on mediators changing functional conditions of oligodendrocytes and precursor cells, respectively (Plemel et al., 2017). However, in the context of ischemic stroke, oligodendrocytes were already discussed regarding their spatial relationships to NVU constituents (Dirnagl, 2012), but they were largely neglected in studies addressing ischemic consequences on the cellular level and concerning their potential role during neuroprotective and restorative processes. Remarkably, oligodendrocyte structures were recently found to be critically affected after experimental focal cerebral ischemia in rats, as light and electron microscopy revealed signs of oligodendrocyte degeneration and swollen axons with demyelination (Han et al., 2017). An impaired oligodendrocyte-axon architecture was also observed in the human brain affected by lacunar infarcts located in the basal ganglia, using comparative analyses of premorbid magnetic resonance imaging and postmortal immunofluorescence labeling (Hinman et al., 2015). Apart from these morphological observations, accumulating evidence emerged concerning the functional link between the endothelium and oligodendrocytes: Mediators like neurotrophic factors were verified to originate from the endothelium, and matrix metalloproteinases as well as the transforming growth factor β were found to be released by oligodendrocytes, overall indicating a critical affection of the BBB integrity (Sandoval and Witt, 2008; Pham et al., 2012; Miyamoto et al., 2014; Seo et al., 2014; Rajani and Williams, 2017). Furthermore, functional interactions were also described between oligodendrocytes and microglia, promoting protective and regenerative reactions in brain pathologies (Peferoen et al., 2014).

These observations strongly support the perspective that oligodendrocytes are more than simple producers of myelin, as they provide numerous cell – cell interactions that allow for instance functional connection to subjacent axons, transmitter exchange and thus maintenance of neuronal activity (Kettenmann and Verkhratsky, 2008; Orduz et al., 2015; Simons and Nave, 2015). As oligodendrocytes originate in the subventricular zone and were found to increase after demyelinating damage of the striatum (Menn et al., 2006), following experimental stroke (Bonfanti et al., 2017) and due to chronic cerebral hypoperfusion (Miyamoto et al., 2015), oligodendrocytes became attraction concerning their ischemic reactions and as a potential target for neuroprotective strategies in the field of ischemic stroke (Arai and Lo, 2009; Zhang et al., 2013; Mifsud et al., 2014; Shindo et al., 2016; Song et al., 2017). However, from the few available studies addressing oligodendrocyte progenitor cells (Ohta et al., 2003; Tanaka et al., 2003) and myelin components such as the myelin basic protein (MBP) after experimental cerebral ischemia, an inconsistent view on ischemia-caused alterations of oligodendrocyte structures has been emerged.

The present study thus investigated diverse oligodendrocyte markers in a translationally relevant – i.e., concerning potential therapeutic interventions typical – time window of 24 h after focal cerebral ischemia in mice. In detail, the oligodendrocyte-specific protein (OSP), known to be located in myelin sheaths of oligodendrocytes and linked to claudin 11 as part of the claudin family involved in tight junction formation (Bronstein et al., 1997; Morita et al., 1999), was targeted in addition to MBP as typical part of the oligodendrocyte myelin sheaths (Nawaz et al., 2013). Further, the neuron-glia antigen 2 (NG2) was analyzed as a marker for oligodendrocyte progenitor cells (Zhu et al., 2008; Orduz et al., 2015). To add a functional perspective, subsequent analyses focused on the 2′, 3′-cyclic nucleotide-3′-phosphodiesterase (CNPase), known to link tubulin with membranes and thus promoting cellular integrity via cytoplasmic microtubuli assembly (Bifulco et al., 2002; Esposito et al., 2008). Referring to current recommendations on preclinical stroke studies (Fisher et al., 2009), this study comprises young and aged animals, and a transgenic background modeling Alzheimer-like alterations to consider relevant co-morbidities (Jellinger and Attems, 2015). The emerging data might help to better understand the role of oligodendrocyte structures during stroke evolution, representing a prerequisite for more elaborated neuroprotective and restorative strategies in acute stroke.

## Experimental Procedures

### Study Design and Induction of Focal Cerebral Ischemia

This study comprised 30 mice from both sexes, whose brains were investigated concerning diverse markers addressing oligodendrocyte structures and the associated vasculature 1 day after focal cerebral ischemia. Considering different age and genetic background, the following groups of animals entered the study: 3-month-old (*n* = 8) and 12-month-old (*n* = 7) Sv129/B6 wild-type as well as 3-month-old (*n* = 8) and 12-month-old (*n* = 7) 3xTg mice, modeling aspects of Alzheimer-like alterations. In detail, the transgenic animals harbored two mutant human transgenes (APP_Swedish mutation_ and tau_P301L_) – driven by neuron-specific Thy1-regulatory elements – and the homozygous knock-in construct presenilin-1_M146V_ (Oddo et al., 2003). All mice were bred by the Medizinisch-Experimentelles Zentrum at Leipzig University, based on breeding pairs kindly provided by Drs. Frank M. LaFerla and Salvatore Oddo (University of California, Irvine, CA, United States).

Focal cerebral ischemia was induced by a right-sided permanent middle cerebral artery occlusion, achieved by a filament-based model according to Longa et al. (1989) with minor modifications. Thereby, a standardized silicon-coated 6-0 monofilament (Doccol Corporation, Redlands, CA, United States) was moved forward from the right external into the internal carotid artery until bending was observed or resistance was felt. For this surgical procedure, mice were anesthetized with etomidate (33 mg/kg body weight i.p.; Hypnomidate, Janssen-Cilag, Neuss, Germany), whereas local anesthesia of the ventral neck was induced via subcutaneous injection of lidocaine (Xylocitin 1%, mibe, Brehna, Germany). During the surgical procedure, the body temperature of the mice was continuously held at around 37.0°C by a thermostatically controlled heating pad equipped with a rectal probe (Fine Science Tools, Heidelberg, Germany). After surgery, mice were placed on a commercially available warming pad until recovery. To ensure sufficient induction of focal cerebral ischemia as study inclusion criteria, during the 24-h observation period animals had to present at least a score of 2 on the Menzies score, naturally ranging from 0 reflecting no deficits up to 4 characterized by spontaneous contralateral circling (Menzies et al., 1992).

All animal experiments were carried out according to the European Communities Council Directive (86/609/EEC, 2010/63/EU), and had been approved by local authorities (Regierungspräsidium Leipzig; reference number TVV 24/10).

### Tissue Preparation

One day after induction of focal cerebral ischemia, mice were deeply anesthetized by a mixture of ketamine (150 mg/kg body weight i.p.; Ketamin-ratiopharm, ratiopharm) and xylazine (15 mg/kg body weight i.p.; Rompun, Bayer, Leverkusen, Germany). The animals were then transcardially perfused with saline, followed by 4% phosphate-buffered paraformaldehyde. Subsequently, the brains were carefully removed from the skulls, post-fixed in the same fixative overnight and equilibrated in 30% phosphate-buffered sucrose. Forebrains were serially cut with a freezing microtome resulting in 10 series of 30 μm-thick frontal sections each. All sections were collected in 0.1 M Tris-buffered saline (TBS), pH 7.4 containing sodium azide and stored at 4°C until histochemical labeling.

### Histochemistry

Prior to all staining procedures, the free-floating sections were extensively rinsed with TBS. Thereafter, non-specific binding sites of tissues for subsequently applied immunoreagents were blocked for 1 h, either with 5% normal goat serum in TBS containing 0.3% Triton X-100 (TBS-T) for the double staining of MBP and biotinylated *Solanum tuberosum* lectin (STL) or otherwise with 5% normal donkey serum in TBS-T. All sections were then incubated for 20 h with mixtures of primary antibodies and biotinylated STL (diluted in the blocking solution) as listed in **Table 1**.

**Table 1:** Double and triple fluorescence labeling.

First marker Primary antibodies	First marker Secondary antibodies^∗^	Second marker Primary antibodies/lectin	Second marker Secondary reagents^∗^	Third marker Primary antibodies/lectin	Third marker Secondary reagents^∗^
Rabbit-anti-claudin 11/OSP (1:400; 241 003, Synaptic Systems, Göttingen, Germany)	Cy3-donkey-anti-rabbit IgG (711-165-152)	Guinea pig-anti-MBP (1:200; 295 004; Synaptic Systems)	Cy2-donkey-anti-guinea pig IgG (706-225-148)		
Rabbit-anti-NG2 (1:100; AB5320; Merck Millipore, Billerica, MA, United States)	Cy2-donkey-anti-rabbit IgG (711-225-152)	Guinea pig-anti-MBP (1:400; 295 004; Synaptic Systems)	Cy3-donkey-anti-guinea pig IgG (706-165-148)		
Rabbit-anti-serum albumin (1:200; 286 003; Synaptic Systems)	Cy2-donkey-anti-rabbit IgG (711-225-152)	Goat-anti-collagen IV (1:100; AB769; Merck Millipore)	Cy3-donkey-anti-goat IgG (705-165-147)	Biotinylated STL (20 μg/ml; B-1165; Vector, Burlingame, CA, United States)	Cy5-streptavidin (016-170-084)
Rabbit-anti-MBP (1:400; 295 002; Synaptic Systems)	Cy3-goat-anti-rabbit IgG (111-165-144)	Biotinylated STL (20 μg/ml; Vector)	Cy2-streptavidin (016-220-084)		
Rabbit-anti-claudin 11/OSP (1:400; 241 003; Synaptic Systems)	Cy3-donkey-anti-rabbit IgG (711-165-152)	Guinea pig-anti-MBP (1:200; 295 004; Synaptic Systems)	Cy2-donkey-anti-guinea pig IgG (706-225-148)	Mouse-anti-CNP 1 (clone 335C6, 1:200; 355 011; Synaptic Systems)	Cy5-donkey-anti-mouse IgG (715-175-151)

*^∗^All fluorescent immunoreagents were obtained from Dianova (Hamburg, Germany) as supplier for Jackson ImmunoResearch (West Grove, PA, United States) and used at 20 μg/ml for 1 h. OSP, oligodendrocyte-specific protein; MBP, myelin basic protein; NG2, neural/glial antigen 2 proteoglycan; STL, *Solanum tuberosum* lectin (= potato lectin); CNP 1, 2′,3′-cyclic nucleotide-3′-phosphodiesterase.*

For double staining of MBP and STL-binding sites, one series comprising each 10th forebrain section from all mice was applied, whereas selected sections were used for all other multiple labeling experiments as summarized in **Table 1**. After several rinses with TBS, immunoreactivities and lectin-binding sites were revealed by incubating the sections for 1 h in a mixture of fluorochromated secondary immunoreagents (at 20 μg/ml TBS containing 2% bovine serum albumin and obtained from Dianova, Hamburg, Germany). All fluorescently labeled sections were finally rinsed with TBS, briefly washed with distilled water, mounted onto fluorescence-free glass slides, air-dried and coverslipped with Entellan in toluene (Merck, Darmstadt, Germany).

In control experiments, the omission of primary antibodies or biotinylated STL resulted in the expected absence of cellular labeling. Additionally, differently fluorochromated immunoreagents visualizing the different markers were switched and thereby produced equal staining patterns.

### Microscopy, Imaging, and Quantitative Analyses

For screening approaches of stained brain sections, a conventional fluorescence microscope (Axioplan, Zeiss, Germany) was used. Pictures from entire sections and from selected regions at various magnifications were made using the microscope Biorevo BZ-9000 (Keyence, Osaka, Japan) or a confocal laser-scanning microscope LSM 510 Meta (Zeiss). Further processing of images including panel generation was carried out with Microsoft PowerPoint (version 2015; Microsoft Corp., Redmond, WA, United States). In some cases, brightness and contrast of micrographs were slightly modified, but without deletion or creation of signals.

For quantitative analyses, from each animal three consecutive MBP/STL co-stained sections were used, while the middle section had to present the most pronounced ischemic lesion. As the three selected sections have had a distance of 300 μm, the focused forebrain area usually comprised an anterior – posterior distance of 600 μm and was located between -0.46 and -1.70 mm from bregma (Franklin and Paxinos, 1997). Using the microscope Biorevo BZ-9000 (Keyence) with control software BZ II viewer (Keyence), in each of the three brain sections per animal, micrographs were taken from 10 regions of interest, while five regions were located on the ischemia-affected hemisphere and five regions on the contralateral hemisphere. Following a shell-like pattern of assumed descending ischemic affection with reference to the ischemic origin as initiated by a filament-based occlusion of the especially proximal parts of the middle cerebral artery, the five regions on the affected hemisphere captured (a) the ischemic core in medial part of the striatum, (b) the striatum *per se* with a slightly more lateral location, (c) the border zone toward non-affected tissue in the medial part, (d) the lateral neocortex involved in the vascular territory of the middle cerebral artery, and (e) the adjacent medial neocortex as part of the anterior cerebral artery territory. These five regions were mirrored to the contralateral hemisphere and served as controls. Images were taken with a 20× objective (visual field: 0.67 mm × 0.47 mm) and a mean shutter speed of 1/120 s. The intensity of the red fluorescence signal originating from Cy3-immunolabeling of MBP was captured in arbitrary units with the software BZ II analyzer (Keyence) for each of the 10 regions per brain section. Further, mean values for each of the 10 regions were built between the three sections per animal, resulting in 10 values for each animal, while each of these values represents one of the addressed region (ischemic core, striatum, border zone, lateral neocortex, and medial neocortex for the ischemia- and non-ischemia-affected hemisphere, respectively). For subsequent analyses, allowing considerations beyond the plain inter-hemispheric comparison, i.e., concerning its reliance on age and the genetic background, differences were calculated between the corresponding regions resulting in intensities of (1) Δ ischemic core, (2) Δ striatum, (3) Δ border zone, (4) Δ lateral neocortex, and (5) Δ medial neocortex.

### Statistical Analyses

All calculations were performed with the IBM SPSS Statistics package version 24.0 (IBM Corp., New York, NY, United States). In addition to descriptive statistics, the Wilcoxon test and the Mann-Whitney *U*-test were used to check for statistical significance between groups, after the overall sample size was explored concerning normal distribution by using the Kolmogorov-Smirnov test and the Shapiro-Wilk test. To consider limitations that may result from the sample size and non-parametric testing, a Monte Carlo simulation was partially added by using a confidence interval of 99% and a sample calculation of 1,000. Further, Pearson correlations were applied to explore interrelations between different parameters. Data are given as means ± standard deviation, unless otherwise indicated. Generally, a *p*-value <0.050 was considered as statistically significant.

## Results

Sufficient focal cerebral ischemia as induced by a filament-based model of middle cerebral artery occlusion was indicated by a relevant neurological deficit in all mice. In detail, mice presented a Menzies score of 3.21 ± 0.64 during the 24-h observation period, while the assessed deficit ranged from 2.0 to 4.0.

### Ischemia-Related Alterations of Oligodendrocyte Markers and Relations to the Vasculature

**FIGURE 1:**
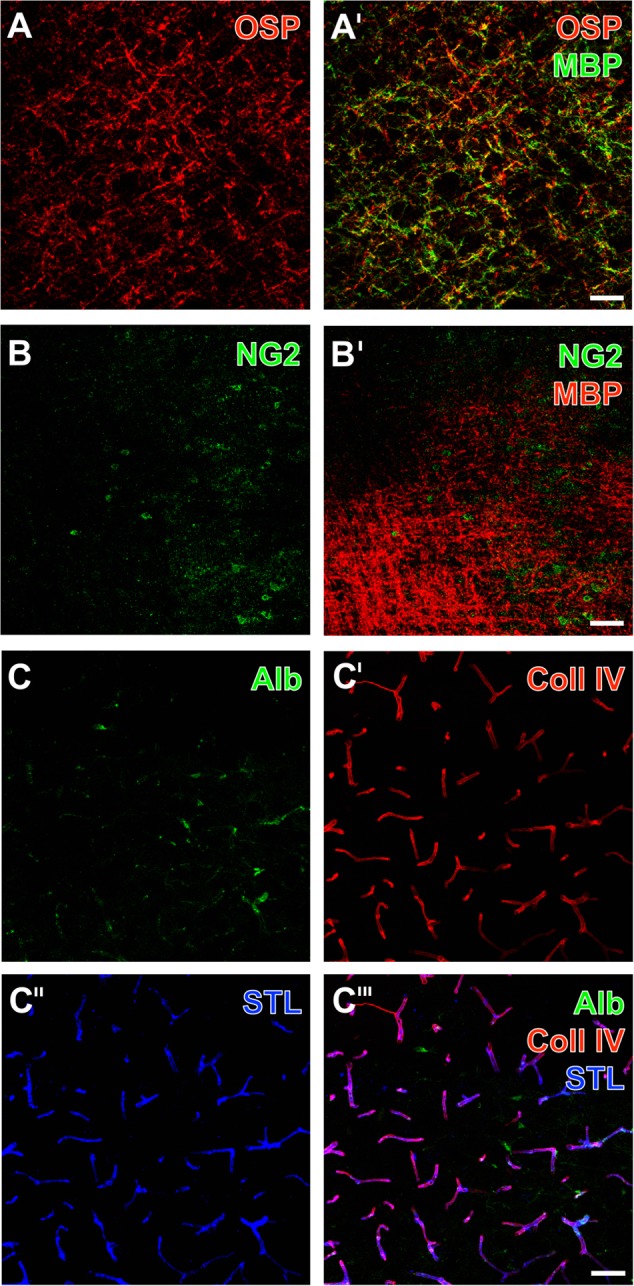
Representative ischemia-related alterations of oligodendrocyte structures and the vasculature in the striatum 1 day after experimental focal cerebral ischemia in 3-month-old (C) as well as in 12-month old mice **(A,B)**, captured by laser scanning microscopy. Concomitant fluorescence staining of the oligodendrocyte-specific protein (OSP, **A**) and the myelin basic protein (MBP, **A′**) as myelin-associated markers exhibited a dense network of dotted and strand-like structures, which appeared visually enhanced due to ischemia. Immunoreactivity of the neuron-glia antigen-2 (NG2) was nearly absent in these tissues **(B)**, while simultaneous detection with MBP clearly showed enhancement of the MBP in the same region **(B′)**. Endogenous serum albumin was found in ischemia-affected areas **(C)** indicating impaired blood – brain barrier integrity, while visualization of the vasculature by collagen IV (Coll IV)-immunostaining **(C′)** and detection of *Solanum tuberosum* lectin (STL)-binding sites **(C″)** appeared robustly with a wide range of overlapping vascular structures **(C″′)**. Scale bars: **A′** (also valid for **A**) = 50 μm, **B′** (also valid for **B**) = 50 μm, **C″′** (also valid for **C–C″**) = 25 μm.

A first set of experiments focused on the detection of oligodendrocyte structures in brain areas affected by 24 h of focal cerebral ischemia. Thereby, OSP-immunoreactivity was found in terms of an inhomogeneous matrix consisting of mostly dotted but also strand-like structures in ischemic areas (**Figure 1A**). Double-labeling of OSP and MBP revealed a strong MBP-immunoreactivity, which appeared more intense and in some part overlapping with the detection of OSP (**Figure 1A**′). However, NG2-immunoreactivity was widely absent in the ischemia-affected brain, and was only observed in terms of some irregularly limited carpets of small dots as well as a few accumulations devoid of a clear cellular or strand-like staining (**Figure 1B**). In contrast, MBP-immunoreactivity in the same region appeared strongly enhanced, building a densely packed matrix of nodular structures and some commutated strands (**Figure 1B**′). Among the three applied oligodendrocyte markers, MBP displayed the visually strongest increase of the immunosignal.

With the intention to investigate the regional patterns of oligodendrocyte structures and the vasculature based on the NVU concept, multiple fluorescence labeling was applied to the ischemia-affected brain tissue. Thereby, detection of endogenous serum albumin allowed the visualization of vessels with inboard albumin as well as some more intense and cloud-like albumin accumulations outside the vessel walls (right part in **Figure 1C**), indicating leaking phenomena due to an ischemia-related reduction in BBB integrity. Further, collagen IV (**Figure 1C**′) as a vascular-associated marker and binding sites of STL (**Figure 1C**″) reliably visualized numerous vessels of different diameters. While merging these patterns, the co-localization of both markers became visible (**Figure 1C**″′), devoid of a clear ischemia-caused vascular affection as regionally demarcated by pronounced serum albumin detection (right part of **Figure 1C**″′).

**FIGURE 2:**
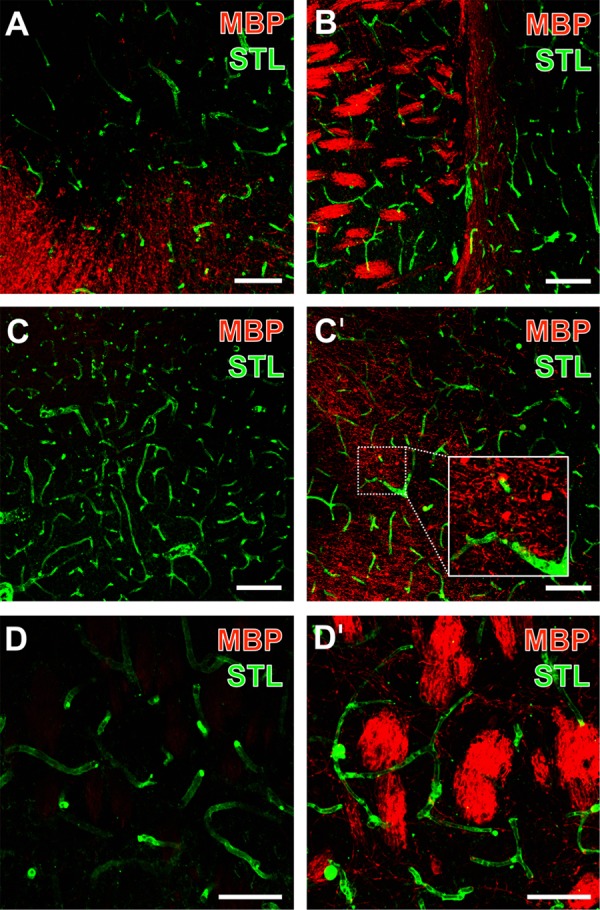
Representative micrographs from double fluorescence labeling of myelin basic protein (MBP) and endothelial STL-binding sites 1 day after focal ischemia in the affected striatum of 3-month-old **(B–D)** as well as 12-month-old mice **(A)**, visualized by confocal laser-scanning microscopy. MBP-immunodetection revealed an increased signal toward the ischemic zone (upper part of **A**, left part of **B**). Inter-hemispheric comparison demonstrated the nearly absent immunoreactivity of MBP **(C)** while vessels were clearly visible as detected by STL **(C)** on the contralateral, non-affected hemisphere. The same striatal region on the ischemia-affected hemisphere exhibited a dense network of MBP-positive structures **(C′)** in close regional association to the vasculature (inset in **C′**). At higher magnifications, densely packed fiber-like structures of MBP were regularly observed in the ischemia-affected striatum **(D′)**, while the contralateral striatum was devoid of these MBP-positive structures **(D)**. Scale bars: **(A)** = 75 μm, **(B)** = 100 μm, **(C,C′)** = 100 μm, **(D,D′)** = 50 μm.

Using the robust lectin-histochemical technique of vessel visualization by STL-binding sites in addition to immunofluorescence labeling of MBP, lectin-positive vessels were found irregularly arranged with respect to the apparently increased immunoreactivity of MBP in areas of ischemic affection (i.e., upper part of **Figure 2A**, and left part of **Figure 2B**). While addressing side-specific aspects, the direct inter-hemispheric comparison revealed a nearly absent MBP-immunoreactivity on the contralateral, non-affected hemisphere (**Figures 2C,D**). In contrast, the ischemia-affected hemisphere exhibited in the more basal area a dense matrix of MBP-positive structures, while especially strands but also cell-like accumulations were arranged in close vicinity to the vasculature (**Figure 2C**′). As a regional peculiarity of the striatum, dense packages of MBP-immunopositive fiber-like structures were noticed in close regional association to vascular elements (**Figure 2D**′).

### Regional Specifications of MBP-Immunoreactivity

**FIGURE 3:**
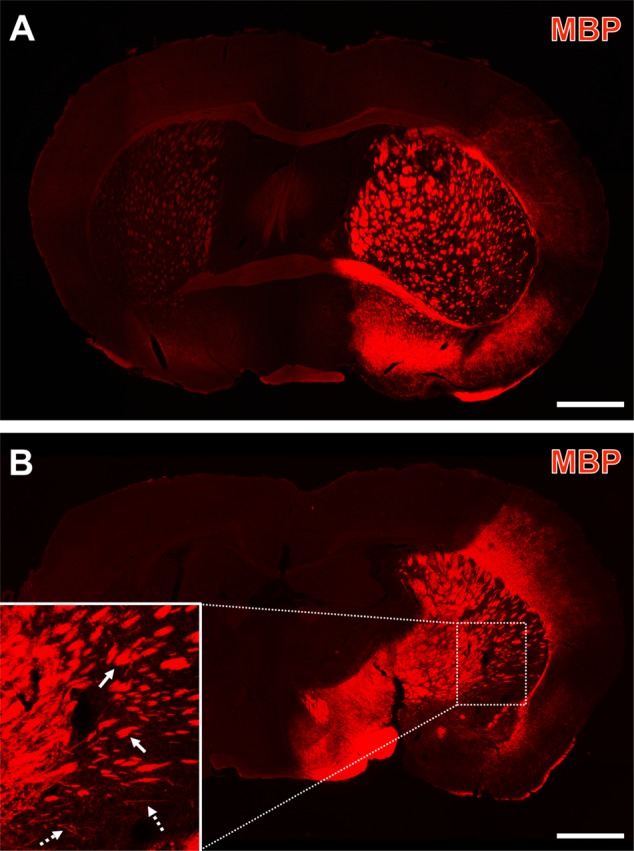
Representative forebrain overview scans from immunolabeled myelin basic protein (MBP) 1 day after focal cerebral ischemia in 3-month-old **(A)** and a 12-month-old mice **(B)**. The enhanced MBP-immunosignal captured the overall territory of the middle cerebral artery (i.e., the ischemia-affected striatum, some thalamic regions and lateral parts of the neocortex), which was occluded by a filament-based model of focal cerebral ischemia. Higher magnification (inset in **B**) indicated dense packages of MBP-immunopositive structures (arrows with plain lines) predominantly in subcortical regions and – toward cortical areas – groups of MBP-positive, mostly parallel arranged strands (arrows with dashed lines). Scale bars: **(A,B)** = 1 mm.

To more closely capture regional aspects of the visually enhanced MBP-immunoreactivity on the ischemia-affected hemisphere, full coronal brain sections were analyzed. Thereby, an enhanced MBP-immunolabeling was found in the nearly complete vascular territory of the middle cerebral artery (**Figures 3A,B**). As regional patterns, carpets of densely packed MBP-positive structures were found to be primarily located in ischemia-affected basal and – to a much lesser degree – neocortical areas. In contrast, subcortical, i.e., striatal regions on the ischemic hemisphere were characterized by dense packages of MBP-immunopositive structures (arrows with plain lines in **Figure 3B**). Remarkably, the conjunction zone between cortical and subcortical regions was characterized by groups of MBP-positive strands that are mostly arranged in a parallel direction (arrows with dashed lines in **Figure 3B**).

**FIGURE 4:**
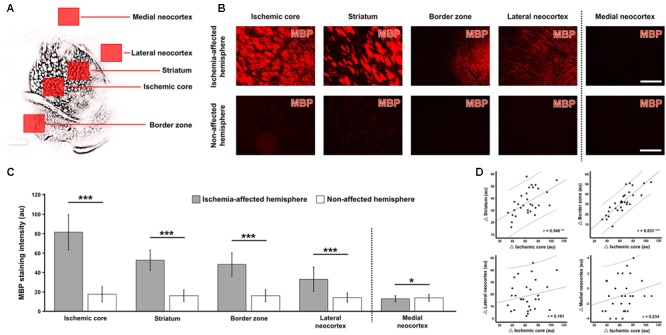
Quantification of myelin basic protein (MBP)-immunoreactivity in different brain areas 1 day after focal cerebral ischemia in mice. Using a shell-like pattern, five regions of interest were arranged on the ischemia-affected hemisphere, as exemplarily shown in panel **(A)** based on a schematic depiction of the respective MBP-immunosignal, while mirrored regions to the contralateral, non-affected hemisphere served as controls. The direct inter-hemispheric comparison revealed a remarkable increase of the MBP-immunoreactivity due to ischemia in all four regions reached by ischemia (**B**, exemplarily captured in a 3-month-old mouse), which was confirmed by quantitative analyses **(C)**. Subsequent correlative analyses of inter-hemispheric differences (Δ values, **D**) indicated simultaneous alterations of the MBP-immunosignal in the ischemic core and in the striatum as well as in the border zone. Scale bars (also valid for all other micrographs) = 200 μm: significance levels: ^∗^*p* < 0.05, ^∗∗^*p* < 0.01, ^∗∗∗^*p* < 0.001; sample sizes: *n* = 29 – 30 for inter-hemispheric comparison **(C)**, and *n* = 29 – 30 for correlation analyses **(D)**. au, arbitrary units.

Subsequent analyses focused on regionally differing MBP-immunosignals. For this purpose, five shell-like arranged regions were used to capture the MBP-immunoreactivity on the ischemic hemisphere (**Figure 4A**) that were compared with five regions mirrored to the contralateral, non-affected hemisphere. Thereby, a visual increase of the immunosignal was observed in the ischemic core, the striatum, the border zone and the lateral neocortex when compared to the contralateral hemisphere (**Figure 4B**). Remarkably, in the medial neocortex as region not belonging to the territory of the middle cerebral artery that is selectively affect by the underlying model of focal cerebral ischemia, a nearly complete loss of the MBP-immunosignal was observed on the ischemia-affected and the non-affected hemisphere (right part of **Figure 4B**). These observations were confirmed by subsequent quantitative analyses on the same regions (**Figure 4C**). In detail, the inter-hemispheric comparison of MBP-immunoreactivity in the overall sample – irrespective of age and the genetic background – revealed a significant increase in intensities due to ischemia in the ischemic core (81.3 ± 18.1 vs. 17.5 ± 7.9), the striatum (52.5 ± 10.4 vs. 16.0 ± 5.8), the border zone (48.1 ± 12.0 vs. 16.1 ± 6.2) and the lateral neocortex (33.0 ± 12.4 vs. 14.1 ± 4.7) (each *p* < 0.001, Wilcoxon test; *p* < 0.001, Monte Carlo simulation). However, a nearly identical immunosignal of MBP became evident in the medial neocortex (12.9 ± 3.1 vs. 13.8 ± 3.6), which, however, achieved marginal statistical significance (*p* = 0.034, Wilcoxon test; *p* = 0.033, Monte Carlo simulation) due to the relatively small standard deviation.

To explore accompanying alterations of the MBP-immunosignal in different ischemia-affected regions, correlation analyses were added involving Δ values between the hemispheres, while using the ischemic core as the area with strongest inter-hemispheric changes as reference (**Figure 4D**). Using the overall sample irrespective of age and the genetic background, these calculations revealed significant interrelations between the changes in the striatum and in the ischemic core (*r* = 0.548, *p* = 0.002; Pearson correlation, explained variance 0.30) as well as between the changes in the border zone and in the ischemic core (*r* = 0.833, *p* < 0.001; Pearson correlation, explained variance 0.69). Given by the positive sign of the correlation coefficient, this finding indicates that the change of MBP-immunoreactivity in the striatum and in the border zone proceeds in the same direction with respect to the ischemic core. However, non-significant interrelations were found between the lateral neocortex and the ischemic core (*r* = 0.191, *p* = 0.321; Pearson correlation) as well as the medial neocortex (*r* = 0.234, *p* = 0.214; Pearson correlation), indicating that the enhanced MBP-immunoreactivity in the lateral neocortex and the slightly decreased immunolabeling in the medial neocortex are not directional associated with a relevant change of the immunosignal in the ischemic core.

### Impact of Age and Genetic Background on MBP-Immunoreactivity

Although qualitative analyses have so far provided evidence for a remarkably robust MBP-immunosignal, a subset of analyses were performed to explore the potential relevance of conditions related to the aging brain and a concomitant Alzheimer-like background. Thereby, inter-hemispheric differences (Δ values) of each region investigated were considered concerning the animal’s age and genetic background. When comparing 3- and 12-month-old mice, Δ values for MBP-immunoreactivity did not differ with respect to the ischemic core (60.1 ± 17.9 vs. 67.9 ± 18.6, *p* = 0.334; Mann-Whitney *U*-test), the striatum (35.5 ± 8.9 vs. 37.5 ± 12.4, *p* = 0.790; Mann-Whitney *U*-test), the border zone (30.4 ± 12.9 vs. 34.0 ± 10.1, *p* = 0.351; Mann-Whitney *U*-test), the lateral neocortex (17.2 ± 9.5 vs. 20.7 ± 14.4, *p* = 0.715; Mann-Whitney *U*-test), and the medial neocortex (-0.9 ± 1.9 vs. -0.9 ± 2.4, *p* = 0.984; Mann-Whitney *U*-test).

Concerning the potential impact of the genetic background, comparative analyses of Δ values for MBP-immunoreactivity in the overall study sample failed to provide significant differences between wild-type and transgenic mice when focusing on the ischemic core (65.8 ± 17.7 vs. 61.7 ± 19.4, *p* = 0.512; Mann-Whitney *U*-test), the border zone (31.8 ± 12.7 vs. 32.1 ± 11.0, *p* = 0.351; Mann-Whitney *U*-test), the lateral neocortex (20.9 ± 12.1 vs. 16.7 ± 12.0, *p* = 0.715; Mann-Whitney *U*-test), and the medial neocortex (-1.0 ± 2.0 vs. -0.8 ± 2.4, *p* = 0.984; Mann-Whitney *U*-test). However, Δ values on MBP-immunoreactivity in the striatum achieved a barely missed statistical significance (40.3 ± 9.2 vs. 32.8 ± 10.7, *p* = 0.050; Mann-Whitney *U*-test).

**FIGURE 5:**
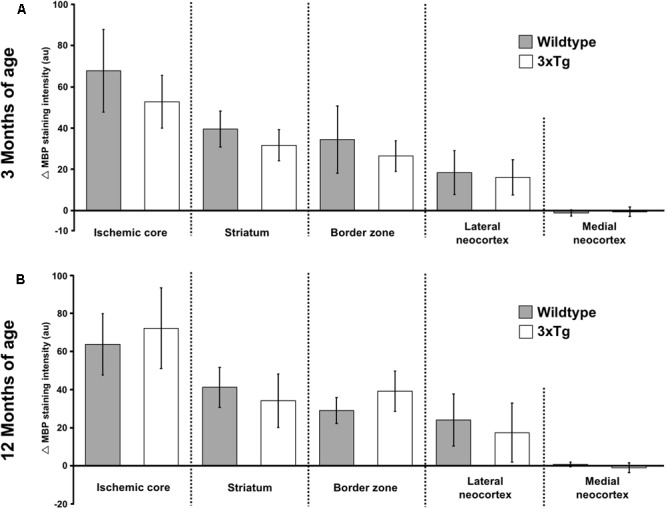
Analyses of the potential impact by age and genetic background on myelin basic protein (MBP)-immunoreactivity. Nearly equal inter-hemispheric differences (Δ values) on MBP-immunoreactivities were observed when comparing wild-type and triple-transgenic mice harboring an Alzheimer-like background. Remarkably, these findings were consistent at different ages (i.e., 3 vs. 12 months of age). Sample sizes: *n* = 6 – 8 per group **(A,B)**. au, arbitrary units.

To allow a differentiated consideration of potential genotype-related effects, inter-hemispheric differences (Δ values) of MBP-immunoreactivity were separately investigated for 3- and 12-months-old animals as well as wild-type and transgenic animals (**Figure 5**). Thereby, means of Δ values did not differ concerning the genetic background in 3-month-old mice (*p*-values ranging between 0.105 and 1.000; Mann-Whitney *U*-test; **Figure 5A**) and in 12-month-old mice (*p*-values ranging between 0.073 and 0.805; Mann-Whitney *U*-test; **Figure 5B**).

### Functional Impact on Altered Oligodendrocyte Structures

**FIGURE 6:**
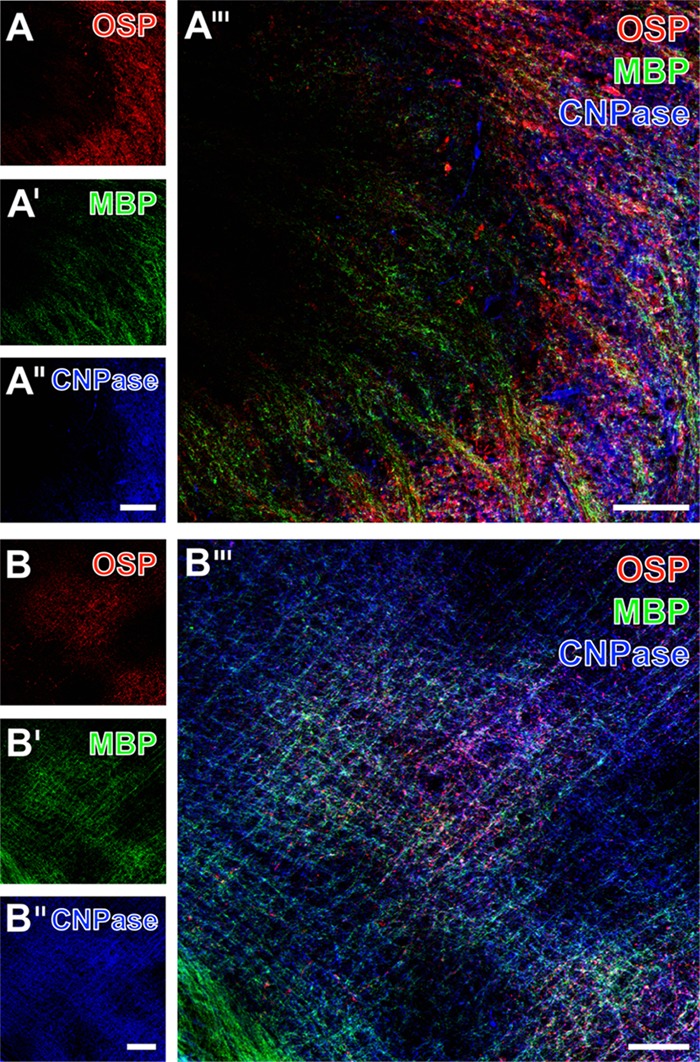
Representative micrographs of a 12-month-old mice 1 day after focal cerebral ischemia demonstrating triple fluorescence labeling of the oligodendrocyte-specific protein (OSP), myelin basic protein (MBP), and 2′,3′-cyclic nucleotide-3′-phosphodiesterase (CNPase). Immunoreactivities for OSP and MBP were enhanced in the ischemic striatum (right part in **A** and **A′**) and the lateral neocortex **(B,B′)**. In a similar manner, the immunosignal of CNPase appeared to be enhanced in the ischemic striatum (right part in **A″**) too, which became even clearer when merging the staining pattern **(A″′)**. Further, an enhanced CNPase-immunoreactivity appeared in the ischemia-affected neocortex except for upper neocortical layers (upper left corner in **B″**), while overlaying structures were predominantly identified in the affected neocortex (middle part of **B″′**). Scale bars: **A″** (also valid for **A** and **A′**) = 100 μm, **A″′** = 50 μm, **B″** (also valid for **B** and **B′**) = 100 μm, **B″′** = 50 μm.

With the intention to explore the functional relevance of altered MBP-immunoreactivity due to ischemia, subsequent immunofluorescence labeling of OSP and MBP was combined with the detection of CNPase, a membrane-bound enzyme that is also involved in maintaining cellular stabilization via microtubule arrangement. At the ischemic border in the mediolateral part of the striatum, allocated and enhanced immunoreactivities for OSP and MBP were observed (**Figures 6A,A′**). Remarkably, CNPase-immunolabeling appeared also enhanced in the ischemia-affected region but nearly disappeared in non-affected areas (left part of **Figure 6A″**). The merging of these staining patterns confirmed the region-specific and in the ischemia-affected area accompanying enhancement of all three oligodendrocyte-associated immunosignals with only a few overlapping structures, while especially MBP and CNPase appeared mainly complementary (**Figure 6A″′**).

In the ischemia-affected neocortex, triple immuno-fluorescence labeling of OSP, MBP, and CNPase revealed a comparable pattern (**Figures 6B–B″′**). In detail, an enhanced immunosignal of CNPase appeared in areas of ischemic affection, accompanied by an enhanced OSP- and MBP-immunoreactivity with the exception of upper neocortical layers (upper left corner in **Figure 6B″**). Detection of MBP and CNPase visualized a matrix of both accumulating immunopositive nodular structures, mostly in a parallel fashion arranged strands (**Figures 6B′,B″**). Compared with subcortical regions, the amount of overlapping MBP- and CNPase-positive structures appeared most pronounced in neocortical areas (**Figure 6B″′**).

## Discussion

The present study aimed to explore histopathological alterations of oligodendrocyte structures caused by experimental focal cerebral ischemia, as oligodendrocytes have been discussed to represent a potential target for advanced neuroprotective strategies in stroke (Arai and Lo, 2009; Zhang et al., 2013; Mifsud et al., 2014; Shindo et al., 2016; Song et al., 2017). Caused by 24 h of experimental focal cerebral ischemia, we found a significantly increased immunoreactivity of MBP with a maximum directly in the core of the ischemic lesion associated with a declining course toward more peripheral areas.

Based on their spatial arrangement, oligodendrocyte structures may be formally seen as part of the NVU (Dirnagl, 2012) as a well-established model capturing diverse cellular affections due to ischemia (del Zoppo, 2008, 2010; Xing et al., 2012). Therefore, this study also captured the spatial association of oligodendrocyte structures with the vasculature, since other constituents of the NVU such as astrocyte endfeet (Hawkes et al., 2013) and microglia (Michalski et al., 2010) were already shown to be affected by ischemia. While focusing on regional arrangements, our study demonstrates oligodendrocyte structures closely associated with the vasculature, strengthening the perspective of an extended NVU model.

Based on recommendations for preclinical stroke studies (Fisher et al., 2009), efforts were made to consider animals of different ages and a transgenic background providing Alzheimer-like specifications as a potential co-morbidity (Jellinger and Attems, 2015). Concerning translational aspects, this study made use of a stroke model with clinical implications, i.e., the wide-ranging affection of the middle cerebral artery territory as induced by proximal and thus large vessel occlusion, which critically affects stroke outcome in the human setting (Friedrich et al., 2015). Addressing potential co-morbid factors, our study provided robust evidence for an independent reaction of oligodendrocyte structures with respect to age and an Alzheimer-like genetic background, thus qualifying these cellular parts as potential targets for future neuroprotective approaches in stroke.

### Oligodendrocyte Alterations Caused by Focal Cerebral Ischemia

From the rather limited well-characterized markers addressing oligodendrocyte components, this study applied two myelin-associated markers, i.e., OSP and MBP (Bronstein et al., 1997; Morita et al., 1999; Nawaz et al., 2013), as well as NG2 known to mark oligodendrocyte progenitor cells (Zhu et al., 2008; Orduz et al., 2015).

In a more general perspective, oligodendrocytes have become of increasing interest due to their potential restorative properties, based on earlier reports that demonstrated the immigration of cells originating from oligodendrocyte progenitor cells originally located in the subventricular zone (Gensert and Goldman, 1997; Zaidi et al., 2004; Menn et al., 2006; Zhang and Chopp, 2009). Consequently, previous research on the role of oligodendrocytes during ischemia mainly emphasized oligodendrocyte precursor cells as usually addressed by the NG2 (Zhang et al., 2013; Song et al., 2017). In detail, Tanaka et al. (2003) investigated the number of oligodendrocyte progenitor cells and mature oligodendrocytes after 90 min of middle cerebral artery occlusion in rats and observed a significant decrease in number within the ischemic core 2 days after reperfusion, while a slight increase of oligodendrocyte progenitor cells was found in the peri-infarct area. Comparable findings were reported by Ohta et al. (2003), who subjected rats to 90 min of focal cerebral ischemia and identified oligodendrocyte progenitor cells in the ischemic penumbra that increase numerically at 3 and 7 days after the ischemic event, while in the ischemic core a decrease was noted. In addition, this study indicated a relevance of age, since 11-week-old mice displayed a significantly greater increase in oligodendrocyte progenitor cells 1 week after the ischemic stimulus than 55- to 70-week-old ones. Consistent data on distribution patterns of oligodendrocytes, i.e., a decrease in the ischemic core and an increase in penumbral areas due to experimental focal cerebral ischemia emerged from Wang et al. (2011). This study also focused on species- and strain-specific features and found that especially stroke-prone spontaneous hypertensive rats exhibited a decrease of oligodendrocyte progenitor cells when compared to Wistar rats. Remarkably, oligodendrocyte progenitor cells were observed in the post-ischemic neocortex when rats had been subjected to an enriched environment, known to improve recovery, which strengthened the hypothesis that these cells may contribute to regenerative issues during the long-term course after ischemia (Komitova et al., 2006). However, in the present study immunoreactivity of NG2 was nearly absent in the ischemia-affected tissue and marked cell bodies or other cellular structures were not clearly detectable. This observation is most likely related to the underlying time point of investigation, as histopathological characteristics were performed at 24 h after ischemia onset, while the referred studies and data from others have captured later time points, usually ranging from 3 days after middle cerebral artery embolization in rats (Claus et al., 2013), until 4 weeks after bilateral common carotid artery occlusion in rats (Chida et al., 2011), and 4 weeks after middle cerebral artery occlusion in rats (Komitova et al., 2006).

Notably, an enhanced OSP-immunoreactivity was found in ischemic brain areas at 24 h after ischemia induction. OSP is known to provide similarities to other elements of the claudin family (e.g., claudin 1 – 8) and was thus discussed to be critically involved in myelin sheaths-associated tight junction formation (Bronstein et al., 1997; Morita et al., 1999; Jiao et al., 2011), suggesting a close functional relationship to BBB maintenance and its impaired function under ischemic conditions. As the present study indicated enhanced OSP-immunoreactivity in ischemic regions also exhibiting BBB impairment as shown by the appearance of endogenous serum albumin in the parenchyma, the perspective that OSP may have a functional impact on BBB integrity is further strengthened. This assumption is also supported by previous data on stroke-related BBB damage. In detail, Krueger et al. (2013, 2015) demonstrated a strong vessel-associated claudin-3- and claudin-5-immunoreactivity concomitantly with an extravasation of the BBB tracer fluorescein isothiocyanate-albumin under ischemic conditions (Krueger et al., 2013, 2015). However, currently the functional relevance of enhanced OSP-immunoreactivity remains to be elucidated. Eligible interpretations might be (1) a reactive up-regulation in terms of an initial step to promote neuroprotection or neuroregeneration by myelin sheaths located in close regional association to the vasculature, or (2) an ischemia-associated degradation of oligodendrocyte these structures with a consecutively enhanced immunosignal.

In the present study, qualitative analyses revealed a strong visually enhanced MBP-immunoreactivity in ischemia-affected areas 24 h after experimentally induced stroke. Conversely, a very recent study has shown a decreasing MBP-immunoreactivity starting at day 3 after endothelin-induced striatal ischemia in rats with a maximum until day 7 (Lima et al., 2016). With an even longer observation period, Chida et al. (2011) investigated the amount of MBP in the corpus callosum of rats subjected to chronic cerebral hypoperfusion as induced by permanent bilateral common carotid artery occlusion and found a consistent decrease at 2 – 12 weeks. These findings and the present data might be relevant for possible translation, as they suggest a highly dynamic and thus modifiable pattern of oligodendrocyte structures emphasizing the associated myelin. This perspective is further strengthened by several human studies targeting oligodendrocytes under diverse brain pathologies. In detail, Bernard et al. (1981) reported already in the early 1980s increased MBP levels in brains of patients suffering from multiple sclerosis. Focusing on brain ischemia, Jauch et al. (2006) investigated blood samples from 359 stroke patients and found a highly significant correlation between the serum concentration of MBP and the ischemic lesion volume as assessed by computed tomography. In a quite smaller sample of stroke patients (*n* = 50), Can et al. (2015) did not find a significant increase in MBP serum levels when compared with controls. An earlier reported, further translationally relevant fact is the measurable MBP in the cerebrospinal fluid of stroke patients (Barry et al., 1991). About 20 years later, Brouns et al. (2010) and Hjalmarsson et al. (2014) also described a significantly increased MBP concentration in the cerebrospinal fluid of stroke patients when compared to controls.

### Regional Characteristics of Affected MBP and Its Functional Relevance

Over the last decades, the penumbra concept became widely accepted describing the evolution of ischemia-related tissue damage over time and thereby separated an ischemic core with definitive loss of cellular integrity from a surrounding penumbral region that may be salvaged in case of vessel re-opening (Astrup et al., 1981; Dirnagl et al., 1999). While considering this concept, the present study applied a shell-like pattern to analyze MBP-immunoreactivity on the ischemia-affected hemisphere in comparison to the contralateral, non-affected hemisphere. Thereby, the qualitative findings on an enhanced immunosignal for MBP at 24 h after experimental stroke were robustly confirmed by quantitative analyses with a maximum of reaction in the ischemic core and a declining course toward more peripheral areas. Further, correlative analyses indicated unidirectional histopathological alterations in the ischemic core and in the striatum as well as in the border zone. As compared with earlier histological studies of oligodendrocytes under ischemic conditions, regional specifications were described for oligodendrocyte progenitor cells as identified by NG2-immunoreactivity (Ohta et al., 2003; Tanaka et al., 2003; Wang et al., 2011), but corresponding data on MBP are still lacking. Solely the study by Gregersen et al. (2001) addressed the regional pattern of MBP after 60 min of middle cerebral artery occlusion in rats by measuring messenger RNAs, and found a disappearance at 24 h within the infarct, while in peri-infarct regions MBP mRNA increased from 24 h until day 7. For the first time, the present study provides region-specific data on MBP-immunoreactivity while considering the well accepted penumbra concept of tissue damage, and indicated that the amount of alteration is directly related to the degree of ischemic affection. Conclusively, the perspective of a region-specific regulation on oligodendrocyte structures with regard to the primary ischemic lesion is further supported by human data. In detail, Brouns et al. (2010) reported a significant increase of MBP levels in the cerebrospinal fluid of patients with subcortical infarcts when compared with those suffering from cortical infarcts. As earlier studies of oligodendrocytes suggested an age-dependency, i.e., a more pronounced reduction of NG2-positive cells in young rats in the ischemic core (Wang et al., 2011), and a stronger increase of these cells in the ischemic penumbra (Ohta et al., 2003), the present study considered also animals of different ages. As non-neuronal elements of the NVU were also described to be susceptible against ischemic stimuli in a co-morbid scenario with neurodegenerative disorders (Zlokovic, 2011; Nelson et al., 2016; Michalski et al., 2017), this study applied a triple-transgenic mouse model exhibiting typical histopathological features of Alzheimer’s disease (Oddo et al., 2003) to consider relevant co-morbidity. Remarkably, immunoreactivities for MBP were robustly affected in the afore-mentioned fashion throughout the ischemic core, the striatum, the ischemic border zone and the lateral neocortex of the ischemia-affected hemisphere, irrespective of age and the genetic background. These data are in line with an earlier report by Badan et al. (2003), who observed a strong and early activation of oligodendrocyte after 70 min of middle cerebral artery occlusion in rats, a feature that is consistent in both 3- and 20-month-old animals. MBP thus appeared as a robust marker for oligodendrocyte affection in the setting of experimental focal cerebral ischemia, at least when focusing on the early phase, i.e., 24 h after ischemia onset.

However, the functional relevance of the observed increase in MBP-immunoreactivity remains further challenging due to the rare data in this field. Previous work indicated a concomitant increase in dendrites and dendritic spines together with a numerical increase of oligodendrocytes as identified by MBP starting at day 7 after permanent middle cerebral artery occlusion in rats (Ueno et al., 2012), which was attributed to regenerative processes. Further, in a model of stereotactically wire knife-lesioning of the entorhino-hippocampal pathway in mice, expression of MBP was investigated until day 35 after the event, and revealed an increase of MBP RNA starting between day 2 and 4, which was interpreted in terms of axonal sprouting (Jensen et al., 2000). To overcome a solely descriptive perspective, qualitative analyses in the present study targeted on the enzyme CNPase to capture microtubule assembly as a prerequisite for cellular integrity. The observed concomitant increase of immunoreactivities for MBP, OSP, and CNPase strongly indicated a simultaneous reaction due to focal cerebral ischemia. However, the apparently enhanced CNPase-immunoreactivity can be interpreted first as part of the early reaction acting toward cellular stabilization, or second as the early initiation of regenerative processes toward axonal outgrowth that requires microtubule formation. From the rare data available here, the second interpretation might be supported by Jing et al. (2013), who focused on the effect of diabetes on stroke. These authors reported a re-appearance of CNPase-immunolabeling in the ischemic lesion started at day 7 after 30 min of middle cerebral artery occlusion in rats (Jing et al., 2013), which was interpreted as remyelination and an element of tissue regeneration.

### Technical Considerations

The presented work was part of a larger study focusing on several components of the NVU in the same stroke model, thus allowing the comparison of its findings with previously published data on ischemia-induced alterations of the vasculature and astrocytic endfeet (Hawkes et al., 2013) and neuronal markers such as the microtubule-associated protein tau (Michalski et al., 2016), as well as the vesicular glutamate transporters 1 – 3 and the vesicular GABA transporter (Michalski et al., 2013). However, this feature also led to a limitation of available brain sections, allowing quantifications only for the MBP-immunoreactivity. Future studies are thus needed to quantitatively investigate changes of CNPase concomitantly with other oligodendrocyte markers, which might allow a more robust interpretation on the functional relevance of observed changes in oligodendrocyte structures.

Three more limitations of the present study need to be addressed: First, further data are necessary regarding biochemical consequences of the ischemic stimulus on the here applied oligodendrocyte markers, as the used immunohistochemical method generally holds the risk of misinterpretation due to the underlying antibody reactions, which might be encouraged in case of more stainable antigens, e.g., due to cellular degeneration, following damaging events. Consequently, subsequent analyses should also include complementary techniques as Western blotting and polymerase chain reactions on oligodendrocyte elements. Second, as tissue damage following stroke was characterized as a multiphasic process (Dirnagl et al., 1999), future studies are needed to characterize oligodendrocyte markers over time. Since the present study was primarily designed while considering translational aspects, a 24-h time point was used to capture histopathological alterations with a delay from ischemia onset that is of relevance concerning therapeutic approaches, i.e., interventions that may target oligodendrocyte structures. Thirdly, this study included mice of both sexes, not considering the perspective of potential hormone-related differences regarding the evolution of stroke and its outcome (Girijala et al., 2017). However, as the sample size at this stage does not allow the exploration of a potential sex-related (factor), future studies would be needed to address this task.

### Summary

This study provides novel evidence for a critical role of oligodendrocyte structures during stroke evolution, and thereby strengthened the perspective of their essential affiliation to the ischemia-affected NVU. Twenty-four hours after experimental stroke, MBP-immunoreactivity robustly increased in ischemic regions with a gradual decline toward more peripheral areas. This feature was regardless of age and the genetic, i.e., Alzheimer-like background. In conclusion, oligodendrocytes and especially myelin-associated proteins such as the MBP were identified to react early after the ischemic event, and thus qualify as potential targets for both neuroprotective and regenerative approaches in stroke. Based on already established treatment strategies, i.e., intravenous thrombolysis and mechanical thrombectomy, interventions supporting oligodendrocyte integrity appear as potential co-treatments with the intention to further improve the long-term outcome of stroke patients.

## Author Contributions

DM carried out the animal experiments. HM generated immunoreagents for this study. WH and ALK performed the histochemical labeling. DM and ALK conducted statistical analyses. DM and WH wrote the paper. DM, JG, and ALK generated the figures.

## Conflict of Interest Statement

HM was employed by the company Synaptic Systems GmbH (Göttingen, Germany). The other authors declare that the research was conducted in the absence of any commercial or financial relationships that could be construed as a potential conflict of interest.
